# HCV antigen instead of RNA testing to diagnose acute HCV in patients treated in the Dutch Acute HCV in HIV Study

**DOI:** 10.7448/IAS.20.1.21621

**Published:** 2017-06-30

**Authors:** Sebastiaan J. Hullegie, Corine H. GeurtsvanKessel, Annemiek A. van der Eijk, Chris Ramakers, Bart J. A. Rijnders

**Affiliations:** ^a^ Department of Internal Medicine, Division of Infectious Diseases, Erasmus MC University Medical Center, Rotterdam, The Netherlands; ^b^ Department of Virology, Erasmus MC, Rotterdam, The Netherlands; ^c^ Department Clinical Chemistry, Erasmus MC, Rotterdam, The Netherlands; ^d^ Department of Medical Microbiology and Infectious Diseases, Erasmus MC University Medical Center, Rotterdam, The Netherlands

**Keywords:** acute hepatitis C, diagnosis, antigen, HIV, HCV RNA

## Abstract

**Introduction**: Affordable and sensitive screening methods for acute hepatitis C (HCV) are necessary to successfully intervene in the current HCV epidemic among HIV-positive men having sex with men. HCV core antigen (Ag) testing has been proven effective in diagnosing chronic HCV-infected patients at low costs. We studied the characteristics of HCV Ag testing in acute HCV-infected HIV-positive patients.

**Methods**: Plasma samples were selected from acutely HCV genotype 1-infected patients treated with peginterferon, ribavirin and boceprevir in the Dutch Acute HCV in HIV Study. The control group consisted of HIV-positive patients with a newly raised alanine aminotransferase (ALT) (>41 U/L) in whom HCV RNA was undetectable and who were tested for HCV Ag. Spearman correlation coefficient between HCV RNA and HCV Ag was calculated together with the sensitivity and specificity of HCV Ag testing at acute HCV diagnosis.

**Results and discussion**: Upon acute HCV diagnosis, HCV Ag was identified in 39 out of 44 patients with detectable HCV RNA levels. In all 23 control patients without detectable HCV RNA in plasma, HCV Ag was undetectable as well. This resulted in a sensitivity and specificity of HCV Ag of respectively 89% (95% CI 75–96) and 100% (95% CI 82–100). The correlation between HCV Ag and HCV RNA was 0.97 (*p* < 0.001) upon diagnosis.

**Conclusion**: The data presented in this study suggest that HCV Ag testing is a sensitive and specific method that can be used in diagnosing AHCV in HIV-infected patients.

## Introduction

There are ongoing epidemics of acute hepatitis C virus (AHCV) infections among HIV-positive men having sex with men and injecting drug users throughout the world [1,2]. AHCV infections are not easily identified because few clinical symptoms characterize the early stage of HCV infection and antibodies are often absent during the early phase of infection [3]. Since 2009 reliable and easily performed automated HCV antigen (Ag) tests are available for the diagnosis of chronic HCV [4]. HCV Ag has shown to correlate well with HCV RNA in chronic HCV but in AHCV infections data are currently sparse [5,6]. Because the use of HCV RNA amplification is a sensitive but expensive method, there is an unmet need for affordable alternatives.

The objective of this study was to determine the performance of the HCV Ag detection as a tool to diagnose AHCV infections in HIV-infected patients.

## Methods

Plasma and serum samples used for this study came from AHCV genotype 1-infected patients treated in the Dutch Acute HCV in HIV Study (DAHHS) [7]. All patients signed a written informed consent and this study was registered under NCT01912495. The availability of samples during the very early stage of infection in this study enabled us to study HCV Ag expression and antibody seroconversion in this well-defined patient group.

An AHCV-infected patient was defined as a patient in whom HCV RNA could be detected and in whom chronic HCV was excluded by testing stored plasma from a preceding outpatient clinic visit for a chronic HIV infection. This included testing for the presence of HCV RNA or HCV antibodies. When this was impossible, a patient was considered to have an AHCV if ALT levels had been normal previously and a negative HCV antibody test had been documented somewhere in the past. Within the DAHHS, screening and baseline (start of treatment) samples were taken within 26 weeks after the calculated transmission date (midpoint between last negative and first positive HCV RNA). For the current study, the first available sample in which HCV RNA was identified is further referred to as “diagnosis”.

To measure the specificity of the HCV Ag test we selected male patients of the Dutch ATHENA cohort with a newly raised ALT (>41 U/L) which was observed during regular ALT screening at least twice per year and who had an undetectable HCV RNA at the time of the ALT elevation [8]. These patients were selected from the HIV outpatient clinic of the Erasmus MC hospital.

All plasma samples were tested for HCV RNA (Cobas CAP/CTM V2, Roche Diagnostics, Almere, the Netherlands or Abbott Realtime M2000, Hoofddorp, the Netherlands), HCV-core antigen (Architect HCV Ag assay, Abbott, Hoofddorp, the Netherlands) and HCV IgG antibody testing was done on serum (Liaison XL anti-HCV, Diasorin, Anderlecht, Belgium). For the HCV Ag test, all data were analysed using the predefined cutoff ratio as previously defined for chronic HCV by the manufacturer (>3.00 fmol/L = positive).

The Spearman coefficient (rho, ρ) was calculated between HCV RNA and HCV Ag. Sensitivity, specificity calculations with 95% confidence intervals based on Wilson score method with continuity correction were performed of HCV Ag against HCV RNA as the gold standard test to diagnose an AHCV infection.

## Results and discussion

The presence of HCV Ag at the time of AHCV diagnosis could be evaluated in 44 out of the 57 patients treated in the DAHHS. Thirteen patients were excluded because insufficient plasma was available. All patients were men, median age was 41 years (interquartile range (IQR) 34–47)) and median CD4 count 675 cells per mm^3^ (IQR 443–788). Median time between last negative and first positive test was 243 days (IQR 209–320). HIV-positive patients with a raised ALT and undetectable HCV RNA were used as control patients (*n* = 23). All controls were men, median age was 41 years (IQR 33–43) and median CD4 count 560 cells per mm^3^ (IQR 390–745).

Upon diagnosis, HCV Ag was observed in 42/44 patients with detectable HCV RNA (95%) of which 39 patients had levels above 3.00 fmol/L (89%). Six patients had an HCV RNA load below 3000 IU/mL. In all of these six patients the Ag loads were below 3.15 fmol/L, with two of them being undetectable. Both patients with an undetectable HCV Ag load had very low HCV RNA loads (221 and 339 IU/mL), low ALT values (31 and 39 U/L), detectable anti-HCV antibodies and both had high CD4 counts with an undetectable HIV load.

The correlation coefficient between HCV Ag and HCV RNA was 0.97 (*p* < 0.001) at diagnosis. Altogether, the sensitivity of the HCV Ag test in this study population was 89% in comparison to HCV RNA (95% CI 75–96). One out of 23 HCV RNA-negative samples was positive for HCV Ag but below the cutoff (0.25 fmol/L) which resulted in a specificity of 100% (95% CI 82–100).

HCV reinfection was diagnosed in 9/44 patients with detectable HCV RNA, based on phylogenetic analysis or a change in HCV genotype. After exclusion of these reinfected patients (in whom antibodies will be present as they remain antibody positive as a result of the previously cured HCV infection), 32/35 RNA detectable patients (91%) were HCV antibody positive at the time of AHCV diagnosis. The three patients with undetectable HCV antibodies upon diagnosis all had high HCV Ag loads (>100 fmol/L). At start of AHCV treatment (<26 weeks after infection) all patients had seroconverted.

The acute phase of an HCV infection is often marked by the absence of IgG antibodies while RNA is detected and the fluctuating viral load during the acute stage [9,10]. This is represented in our data set because 21% of the patients had an HCV RNA load between 15 and 10.000 IU/mL. Remarkably, we found an antibody seroconversion rate of 91% after 121 days of infection. This is in contrast to a previous large study that found a substantially lower seroconversion rate [10]. This early seroconversion in the total group of acute HCV-infected patients suggests that antibodies are still of use in diagnosing acute infection.

Unfortunately, the DAHHS included only genotype 1 patients (98% 1a), which is the genotype that is present in approximately 60–80% of AHCV-infected HIV-positive patients in Europe [1,9,11]. We cannot draw definite conclusions on the use of HCV Ag testing for the detection of other HCV genotypes. However, in previous studies on the use of HCV Ag in chronically HCV-infected patients, no difference between HCV genotypes was reported [12,13]. Additionally, based on our results, the effect of HIV infection on the detection of HCV Ag during the acute phase cannot be established. Therefore we cannot conclude whether the HCV Ag test will perform similarly in high-risk HIV-negative patients. A recent meta-analysis on the performance of HCV Ag testing between HIV-positive and -negative patients in chronic HCV-infected patients unfortunately lacked sufficient power to draw conclusions [14].

If the goal of HCV detection is to reduce transmission or eliminate the virus, a test with 100% sensitivity would be ideal. The sensitivity of the HCV Ag test could be improved when the cutoff ratio of 3.00 fmol/L that was validated for the use in patients *chronically* infected with HCV would be ignored and every patient with a detectable signal would be confirmed with HCV RNA. This would result in an increased sensitivity of the HCV Ag test of 95% but also a small decrease of specificity to 96% and thus a small increase in unnecessary HCV RNA testing to confirm the positive HCV Ag test. Thus, in a context where avoiding a delay in the diagnosis of an acute HCV infection would be crucial, the use of any level of HCV Ag detection as the cutoff for HCV RNA confirmatory testing may be preferable. Also in case of a patient with high-risk factors, a raised ALT and a completely undetectable HCV Ag signal, a treating physician should consider HCV RNA testing to prevent a missed AHCV infection and thereby ongoing transmission.

Finally, given the much higher cost of HCV RNA testing, the use of an HCV Ag test (and if positive followed by HCV RNA testing) is probably more cost-effective to diagnose or exclude an acute HCV infection in an HIV-positive patient with a new ALT elevation.

## Conclusions

This study suggests that HCV Ag testing is a sensitive and specific method that can be used for the purpose of diagnosing AHCV in HIV-infected patients.
